# Transition age youth mental health: addressing the gap with telemedicine

**DOI:** 10.1186/s13034-022-00444-3

**Published:** 2022-02-02

**Authors:** Susheel K. Khetarpal, Lauren S. Auster, Elizabeth Miller, Tina R. Goldstein

**Affiliations:** 1 University of Pittsburgh School of Medicine, 3550 Terrace Street, Pittsburgh, PA 15213 USA; 2 Department of Psychiatry, University of California San Francisco, San Francisco, CA USA; 3 Division of Adolescent and Young Adult Medicine, UPMC Children’s Hospital of Pittsburgh, Pittsburgh, PA USA; 4 Department of Psychiatry, Western Psychiatric Hospital, Pittsburgh, PA USA

**Keywords:** Adolescent, Transition age youth, COVID-19, Telemedicine, E-therapy, Transition to adult services, Health care policy, Health care access

## Abstract

Transition age youth (TAY), a demographic spanning ages 15–26, navigate a myriad of developmental transitions, ranging from identity formation and intimate relationships to substance use. Unfortunately, many young adults continue to have a dearth of mental health services and programing tailored to their unique developmental needs. Moreover, the systems of care in place are generally designed for treating traditional pediatric and adult patients but not ideally suited to meet the needs of TAY. Given the additional stressors from the COVID-19 pandemic, TAY are now, more than ever, in need of routine mental health care. We posit that the rapid expansion of telemedicine programming developed in response to the pandemic could be beneficial in mitigating this historic gap in care. In this commentary, we call on mental health providers and researchers to expand and invest in the growing number of telemedicine interventions and programming for this population so that TAY can begin to receive the care they so desperately need.

## Introduction

Transition age youth (TAY) encompasses a broad demographic spanning from older adolescence to young adulthood (15–26 years old). This population includes diverse subgroups of youth, who vary across broad domains, including but not limited to physical and behavioral health diagnoses, education, socioeconomic status, and social determinants of health.

What unites all TAY, however, is that they experience a myriad of psychosocial transitions [1–3]. These include gaining and maintaining independence, identity formation, exploring sexuality and relationships, post-secondary education, career development, and engaging in behaviors that have potential for poor health outcomes (e.g., substance use, violence, self-harm) [1, 2]. This developmental stage also appears particularly vulnerable to new-onset of mental health disorders, including mood, psychotic, and substance use disorders as well as suicide ideation and attempts [1–3]. Moreover, the mental health needs of TAY have escalated during the COVID-19 pandemic, with social distancing policies disrupting daily routines, social supports, relationships, employment, and typical coping strategies including connections with friends [4–7].

Despite this ever-clear need for behavioral health care, TAY experience numerous unique barriers to receiving mental health resources, including rigid cutoffs for pediatric services ending between ages 18–21, lack of systematic transition from school-based services after graduation from high school, unfamiliarity with adult mental health providers and resources, difficulty navigating transitions from pediatric to adult systems of care, and greater perceived responsibility and ownership for medical decisions [1–3]. These obstacles reinforce the already low rates of mental health care seeking as well as treatment receipt and retention among TAY, maintaining the gap in mental health care for this population [1–3]. Unfortunately, inadequate quantity and access to mental health resources during this critical developmental period can impact long-term trajectories of functioning [1]. By supporting young people with resources they need to navigate this transition to adulthood, we can mitigate against adverse health outcomes and, in turn, promote personal achievement and thriving.

Recognizing the unique needs of TAY nearly a decade ago, Wilens and Rosenbaum concluded in their clinical perspective piece titled *Transitional Aged Youth: A New Frontier in Child and Adolescent Psychiatry* that “child and adolescent psychiatrists are uniquely situated for the direct clinical care of TAY and to oversee the care of TAY in innovative programs” [3].

Despite these recommendations, there remains a dearth of mental health programs designed for TAY across a variety of medical disciplines (i.e., family medicine, pediatrics, adult medicine, and child psychiatry). Those documented in the literature, while demonstrating feasibility, primarily focus on subgroups within the broader TAY population (e.g., TAY with Autism Spectrum Disorder) [8] and often rely on school- and college-based care structures [8, 9], which are not applicable to all TAY. Furthermore, racial, ethnic, and geographic disparities in TAY mental healthcare access and outcomes remain, with minoritized youth often not receiving care [1–3]. For example, even though adolescents living in neighborhoods with concentrated disadvantage often receive mental health supports in public grade schools, this source of services and supports drops off dramatically when these youth graduate high school, especially for those who move into the workforce directly rather than continuing with higher education[1–3]. Recognizing the structural inequities by race, gender, and access to healthcare among this population, coupled with the unique experiences and stressors of young adulthood, more inclusive and equitable mental health interventions are needed urgently.

With the onset of the COVID-19 pandemic, telemedicine has become more ubiquitous and may be one strategy for reaching TAY more broadly [5, 6], especially given this population’s general comfort using social media and technology to connect, communicate, and create [10, 11]. In response to the COVID-19 pandemic, health systems have expanded virtual and app-based services, including but not limited to hotlines, video-based therapy, peer-support groups, medical apps, and ecological momentary interventions [6, 10–12]. Beyond accessibility, there is growing support that telehealth programs may provide equivalently effective care for adolescents across a variety of mental health conditions [11, 13]. For example, KANOPEE, a free smart phone app developed in France to help youth with sleeping behaviors made worse in the context of COVID-19, is a feasible telehealth intervention for adolescents addressing topics relevant to their health and well-being [14]. Many other examples of COVID-relevant telehealth programs for pediatric mental health exist and continue to be studied for their acceptability and effectiveness [15].

Within a social justice and health equity framework, telehealth could improve access to and receipt of care by reaching TAY with geographic barriers to care as well as those without access to institutions where care is typically delivered [10, 13]. Programs could also be paired with a referral system to support youth requiring specialized treatment. For example, from a prevention perspective, implementing promising transdiagnostic interventions that target common stressors (e.g., sleep, interpersonal relationships, transition to independence) could assist a larger number of minoritized youth and connect them with mental health clinicians and resources for more specialized care as necessary [16].

Critical to the success of virtual mental healthcare for TAY will be identifying barriers to telemedicine and developing subsequent solutions. Notable barriers include limited patient privacy and confidentiality, lack of co-location within an interdisciplinary team, lack of appropriate technology among patients (especially those living in poverty), loss of connectedness between providers and patients, language barriers, and high attrition rates, to name a few [17, 18]. While telehealth programming to date has largely not been able to rectify these shortcomings, many promising strategies warrant continued investigation. Proposed solutions to the above-mentioned barriers include the creative use of video conferencing features, bolstering an internal referral system, individualizing sessions when possible, providing technical support or hybrid care formats, and integrating interpretation services into the telemedicine platform [18, 19]. Such solutions could be similarly applied to virtual mental health programming for TAY.

Another growing challenge to rapid expansion of telemedicine for TAY mental health services in the United States is lack of reliable, adequate insurance coverage, and reimbursements [20]. The relaxation of this barrier in March 2020 for provision of clinical care during the COVID-19 pandemic allowed behavioral health providers to reach their patients and provide continuity of care [20]. Now, numerous states are debating when this emergency provision will end, and a standardized approach for how to cover the ongoing, tremendous demand for telehealth has not yet emerged at the federal level. We advocate for continuing reimbursement for and reducing restrictions on telemedicine services, as patients continue to seek mental health care in the ongoing aftermath of COVID-19.

## Conclusion statement

Although Wilens and Rosenbaum coined transition aged youth (TAY) as the “new frontier” nearly a decade ago [3], TAY continue to lack adequate and developmentally appropriate access to mental health care. We posit the rapid expansion of telemedicine in the context of the COVID-19 pandemic provides mental health providers across numerous disciplines a unique opportunity to address this gap, work towards more equitable care, and better equip our young adults to prosper and thrive.

## Data Availability

Not Applicable (commentary article).
